# *Gaultheria trichophylla* (Royle): a source of minerals and biologically active molecules, its antioxidant and anti-lipoxygenase activities

**DOI:** 10.1186/s12906-016-1511-4

**Published:** 2017-01-03

**Authors:** Fiaz Alam, Qazi Najam us Saqib, Mohammad Ashraf

**Affiliations:** 1 Department of Pharmacy, COMSATS Institute of Information Technology, 22060 Abbottabad, Pakistan; 2 Department of Biochemistry and Biotechnology, Islamia University of Bahawalpur, 63100 Bahawalpur, Pakistan

**Keywords:** Antioxidant, Anti-inflammatory, Himalayan snowberry, Phenolics, Minerals, HPLC

## Abstract

**Background:**

*Gaultheria trichophylla* (Royle) is used as food and for treating many ailments in folk medicine especially against inflammation. The purpose of this *in vitro* study was to evaluate the ability of extracts of *G. trichophylla* as anti-oxidant and anti-inflammatory agent and for its mineral contents.

**Methods:**

Powdered plant material (100 g) was extracted with 100 ml each of methanol, chloroform, and n-hexane using soxhlet extractor. Antioxidant activity of methanol extract was assessed by DPPH radical scavenging and FRAP assays. Determination of enzyme inhibition activity was determined using 5-LOX inhibitory activity. Total phenolic and flavonoids contents were measured by Folin-Chicalteu and colorimeteric methods respectively. Minerals and heavy metals contents were determined using Atomic absorption spectrophotometer. Qualitative HPLC analysis were performed using some standard phenolic compounds.

**Results:**

The highest phenolic (17.5 ± 2.5 mg GA equivalent/g) and flavonoids (41.3 ± 0.1 mg QE equivalent/g) concentrations were found in methanol extract, which also showed more scavenging activity of 1, 1-diphenyl-2-picrylhydrazyl and ferrous reducing power with IC_50_ = 81.2 ± 0.2 and IC_50_ = 11.2 ± 0.1 μg/ml, respectively. The methanol and chloroform extracts showed best inhibition of 5-lipoxygenase enzyme with 90.5 ± 0.7% and 66.9 ± 0.1% at 0.5 mg/ml, respectively. *G. trichophylla* extract was also evaluated for mineral contents (K, Na, Ca, Mg, Fe, and Cu), and for chemical profiling of heavy metals (Cr, Pb, Cd, Co, Zn, Ni and Hg).

**Conclusion:**

Our current findings suggest that this plant is good source of minerals and concentration of all heavy metals were within permissible limits. The results revealed that this ignored plant has great pharmaceutical and nutraceutical potential.

## Background

The free radicals and reactive oxygen species (ROS) leads to the formation of harmful chemical compounds and have an important role in the management of health and disease and is therefore producing a medical revolution [1]. Research suggests that free radical damage to cells leads to the pathological changes associated with aging and therefore have an increasing number of diseases or disorders, as well as the aging process itself, demonstrates link either directly or indirectly to these reactive and potentially destructive molecules [2].

The antioxidants through their free radical scavenging property can delay or inhibit cellular damage. The antioxidants are usually being low-molecular-weight and can interact with free radicals and terminate the chain reaction before the important molecules are damaged. Antioxidants are produced in the body and others are found in the diet [3]. The intake of natural antioxidants has been reported to reduce risk of cancer, cardiovascular diseases, diabetes and other diseases associated with aging [4]. In many cases, it is concluded that antioxidants modulate the pathophysiology of chronic inflammation up to some extent [5]. In view of increasing risk factors of synthetic phenolics to human health, there has been a trend globally toward the use of medicinal and dietary plant substances as therapeutic antioxidants. Many antioxidant compounds, naturally occurring in plant sources have been identified as free radical or active oxygen scavengers [6]. Lipoxygenases (LOX) are enzymes are correlated with inflammatory and allergic reactions because of the formation of the leukotrienes (LTs) [7]. Increased levels of leukotrienes could be observed in the pathological conditions of asthma, psoriasis, allergic rhinitis, rheumatoid arthritis and colitis ulcerosa. The production of LTs can be prevented via inhibition of the lipoxygenase pathway [8]. The drugs having ability to inhibit LOX isoforms and/or their biologically active metabolites can be useful in cancer treatment [9].

Other than medicinal values the plants and their polyphenolic compounds have become the focus of current nutritional interest due to their health-promoting effects [10]. Because of the increasing interest in traditional medicinal products, it is important to determine whether they are safe for consumption. Levels of toxic elements such as As, Cu, Cd, Hg and Pb in the plant samples must be determined. Some common elements such as K, Na and P are essential for health and the quantification of these elements is important for nutritional purposes [11–13].

Many species of plants have been successful in absorbing metal contaminants which are essential for plant growth (Fe, Mn, Zn, Cu, Mg, Mo, and Ni). Some metals with unknown biological function (Cd, Cr, Pb, Co, Hg) can also be accumulated [14]. Arsenic, mercury, and lead are contaminants found in the environment which are notoriously toxic to man and other living organisms. Investigation of such heavy metals is necessary before the adjustment of the final recommended doses of the plant to avoid toxicities [15]. Therefore, there is an urgent need for quick assessment of these heavy metals in medicinal plants to control the level of contaminants in herbal raw materials [16].

*Gaultheria* (Ericaceae) consists of over 1700 species. *Gaultheria trichophylla* (Royle) is native to the Himalayas and commonly known as Himalayan Snowberry [17]. It is more distinct due to its blue color berries which are eaten as refreshing food from the local community and red to pink color flowers. The small green leaves are approximately 3 – 7 mm in length. This plant is furnished with setae and found in cold and lofty situations of the mountains [18]. Research work on other species of this plant indicates anti-inflammatory, [19], antibacterial, [20] and anti-arthritis [21] activities. Other species of the genus *Gaultheria* like *G.yunnanensis*, *G. fragrantissima* and *G. procumbens* are used in traditional medicine to treat arthritis in China, India, America and Canada. Gaultherin a natural salicylate isolated from *G.yunnanensis* possess analgesic and anti-inflammatory activity [22]. The phytochemical investigation of the species investigated reported to contain methyl salicylate, diterpenoids, acids, dilactone, alkaloids and other glycosides [23].

The present approach was based on the fact that *Gaultheria trichophylla* was described as being used as anti-inflammatory in traditional medicines, therefore it was evaluated *in vitro* for antioxidant and lipoxygenase inhibitory activities, and to determine its potential as a safe functional food and alternative medicine.

## Methods

### Plant material

The *Gaultheria trichophylla* plant was collected from Kaghan valley, District Mansehra, KPK, Pakistan, in the month of November, 2013. After authentication by taxonomist, Professor Dr. Qazi Najum us Saqib, voucher specimen (CTPHM-GT01, 13) was deposited in the herbarium of the Department of Pharmacy, COMSATS Institute of Information Technology, Abbottabad. After washing with water the whole plant was dried in shade at room temperature. The dried material was ground to a coarse powder. The powder drug was stored in air tight and light resistant container before extraction.

### Extract preparation

The powder material (100 g) was extracted with each of methanol, chloroform and n-hexane using soxhlet extractor for 20 h each. It was filtered through a Whatman Grade-I filter paper. The filtrate was evaporated on a vacuum rotary evaporator under reduced pressure at 40 ^°^C. Extractive yield (percent) of the methanol (Gt. MeOH), chloroform (Gt. Chlor) and for n-hexane (Gt. Hex) was 21.85%, 16.35% and 7% respectively.

### Chemicals

1, 1-diphenyl, 2-picryl hydrazyl (DPPH), Ascorbic acid (Vitamin C), Gallic acid, Quercetin, tetrazolium chloride, Folin Ciocalteu’s reagent, 5-LOX enzyme from soybean, KH_2_PO_4_ buffer, and Baicalein were all purchased from Sigma-Aldrich, St. Louis, MO, USA. DMSO dimethyl sulphoxide, and Methanol (analytical) were purchased from Merck, Germany. HClO_4_, H_2_SO_4_, and Standards of Na, K, Ca, Fe, Pb, Mn, Cr, Zn, Cd, Co, Hg and Ni procured from Merck, Germany.

### Determination of total phenolic contents (TPC) and qualitative HPLC analysis

TPC of methanol extract of *Gaultheria trichophylla* was determined by the Folin-Chiocalteu method [24–26]. The modification made in the method was to use gallic acid as a standard phenolic compound. The extracts were diluted with distilled water to a known concentration in order to obtain the readings within the standard curve range of 0.0 to 600 μg of gallic acid/ml. 250 μl of diluted extract or gallic acid solution was mixed with 1 ml of distilled water in a test tube followed by the addition of 250 μl of Folin-Chiocalteu reagent. The samples were mixed well and then allowed to stand for 5 min at room temperature and allowed to complete a reaction with the Folin-Chiocalteu reagent. Then, 2.5 ml of 7% sodium carbonate aqueous solution was added and the final volume was made up to 6 ml with distilled water. The samples were then incubated for 90 min. The absorbance of the resulting blue color solution was measured at 760 nm using spectrophotometer. The result was expressed as mg of gallic acid equivalents (GAE)/g of extract by using an equation that was obtained from standard gallic acid graph. All the experiments were conducted in triplicates.

High-performance liquid chromatography (HPLC) with autosampler (PerkinElmer series 200, PerkinElmer, Waltham, MA), UV–visible detector, column (SUPELCO) C18, 25 cm, 4.6 mm^2^, particles diameter 5 mm was used. Samples were analyzed using Total ChromWork Station software (SAS Inc.,Cary, NC), version 6.3 with LC Instrument control.

Hydroxy benzoic acid (HBA), Catechin (CA) and epicatechin (ECA) were purchased from Sigma (St. Louis, MO), and quercetin (QU) and kaemferol (KA) from Fluka (Seelze, Germany). All the HPLC grade solvents for elution and sample preparation were purchased from Merck (Darmstadt, Germany).

The gradient elution of solvent A [water/acetonitrile/formic acid (95:5:0.1, *v*/*v*/*v*) and solvent B (acetonitrile/formic acid (100:0.1, *v*/*v*) had a significant effect on the resolution of compounds. Solvent B was increased to 50% in 4 min and subsequently increased to 80% in 10 min at a flow rate of 1 mL per minute. Detection wavelengths were 250, 310, 280, and 360 nm. Injection volume was 10 ml for each sample and reference. Run time was 15 min. Before injection, sample (20 mg) and reference were dissolved in 70% (*v*/*v*) aqueous methanol (10 mL) and filtered through a 0.45 -mm membrane filter (Millipore,Billerica, MA) (Michel et al. 2014).

### Determination of total flavonoid contents

The total flavonoid contents of *G.trichophylla* was determined by using of a modified colorimetric method described previously [27]. The dried plant extract (25 mg) was ground in a mortar with 10 ml 80% methanol. The homogenous mixture obtained was allowed to stand for 20 min at room temperature. Mixture was filtered through filter G4. An aliquot of 0.4 ml of filtrate was mixed with 0.6 ml distilled water, 5% NaNO_2_ solution (0.06 ml). The mixture was allowed to stand for 5 min at room temperature. After 6 min 10% AlCl_3_ solution (0.06 ml) was added to the mixture. 1 N NaOH (0.4 ml) and 0.45 ml distilled water were added immediately, to the mixture and allowed to stand for another 30 min. Absorbance of the mixture was determined at 510 nm and Quercetin was used as standard compound for the quantification of total flavonoid content. All values were expressed as milligram of Quercetin equivalents per 1 g dry weight. Data was recorded as mean ± SD for three replicates.

### DPPH radical scavenging assay

DPPH radical scavenging activity was evaluated as described by [28] with some modification. Ten μl of test solution was added in 96-wells plate followed by the addition of 90 μl of 100 μM methanolic DPPH solution in a total volume of 100 μl. The contents were mixed and incubated at 37 °C for 30 min. The reduction in the absorbance was measured at 517 nm using Synergy HT BioTek® USA microplate reader. All experiments were carried out in triplicates. For the determination of IC_50_ values, test solutions were assayed at various dilutions i.e. 0.5, 0.25, 0.125, 0.0625, 0.0313, 0.015 mM. Data obtained was computed using the computer software Statview version 5.0. Ez-fit software. Standard compounds used were quercetin and Vitamin C (as positive control). The percent inhibition by sample exposure was determined by comparison with a DMSO treated control group. The obtained data were used to determine the concentration of the sample required to scavenge 50% of the DPPH free radicals… The decrease in absorbance indicates increased radical scavenging activity which was determined by the following formula.

Inhibition (%) = (Absorbance of control – Absorbance of test solution/Absorbance of control) × 100.

### Ferrous reducing antioxidant power (FRAP)

This assay uses antioxidants to reduce ferric ion in a complex with tripyridiltriazine (Fe3 -TPTZ) to an intense blue ferrous (Fe^2^) complex that develops an absorption maximum at 700. FRAP values are obtained by comparing the absorbance change at 700 nm in test reaction mixtures with those containing ferrous ions in known concentrations. Hence, this test measures the ability of a sample to reduce ferric ion complex [29].

Ferrous Reducing antioxidant power activity of extracts was determined according to the method of [30] with some additional changes. In the well of 96 microplate 25 μl of test sample was mixed with 25 μl of phosphate buffer (pH 7.2) and then added 50 μl of 1% potassium ferricyanide solution into the mixture. This mixture was incubated for ten min at 50 °C then 25 μl of 10% trichloroacetic acid (w/v) solution and 100 μl distilled water was added into it then measured the absorbance at 540 nm micro plate reader and was taken as pre read value. At last, 25 μl of freshly prepared 0.2% ferric chloride (FeCl_3_) solution was added into the mixture and measured the absorbance at wavelength of 700 nm on a micro plate reader. Quercetin was used as standard. For the determination of IC50 value, sample solution was assayed with different concentration until it reaches the %inhibition of 50. The ferrous reducing power was measured by using the formula as given below;

%inhibition = (absorbance of sample/absorbance of control) × 100

### 5-LOX Assay

5-LOX activity was assayed according to the method [31] with slight modifications. All extracts assays carried out in triplicate. A total volume of 200 μl contained 140 μl KH_2_PO_4_ buffer (100 mM, pH 8.0), 20 μl test compound and 15 μl purified LOX enzyme (127 units per well). The contents were mixed and pre-read at 234 nm and pre-incubated for 10 min at 25 °C. The reaction was initiated by the addition of 25 μl substrate solution. The change in absorbance was observed after 6-10 min at 234 nm. Baicalein was used as a positive control. The determination of IC_50_ values were determined at various dilutions i.e. 0.5, 0.25, 0.125, 0.0625, 0.0313, 0.015 mg. Data obtained was computed on Ez-fit software. The decrease in absorbance indicates increased radical scavenging activity which was determined by the following formula;

Inhibition (%) = (Abs. of control – Abs. of test solution/Absorbance of control) × 100.

Where,

Absorbance of control = Total enzyme activity without inhibitor,

Absorbance of test = Activity in the presence of test compound

### Determination of minerals

Minerals (Na, Ca, Mg, Fe, K, Zn, Mn, and Cu) were measured by using an atomic absorption spectrophotometer (Perkin Elmer AAnalyst700, USA). Before analysis the samples were digested in mixture of H_2_SO_4_, HNO_3_ and HClO_4_. All determinations were done in triplicate. The minerals were expressed as mg/100 g of fresh weight [32].

### Determination of heavy metals

Heavy metals (Ni, Pb, Hg, Si, Co, Cr, As and Cd) contents analysis in *Gaultheria trichophylla* are determined according to method of [33] using an atomic absorption spectrophotometer (Perkin Elmer AAnalyst700, USA). Standards of Ni, Pb, Hg, Si, Co, Cr, As and Cd were used as reference analytes for quantitative estimation of heavy metals as well as accurate calibration of each analyte. The standard stock solutions (1000 ppm) were diluted to obtain working standard solutions ranging from 1 ppm to 10 ppm and stored at 4 °C. An acidity of 0.1% nitric acid was maintained in all the solutions. A calibration curve was plotted between measured absorbance and concentration (ppm). All the samples were analyzed in triplicate. Samples were digested in 20 ml mixture of concentrated acids (nitric and perchloric acid in 9:1 ratio) for 3 h in a water bath maintained at 70 °C for dissolving the contents until a clear brownish solution was obtained using wet digestion method. After cooling, these solutions were reconstituted with deionized autoclaved water to 20 ml. Each sample was filtered using whatmann filter paper (pore size 0.45 μ, Axiva) and stored in closed acid-washed glass vials.

## Results

### Determination of phenolic and flavonoid contents

The *Gaultheria trichophylla* was evaluated for their phenolic contents by Folin-Chiocalteu assay.

The methanol extract showed the highest phenolic content 17.58 ± 2.51 mg GAE/g of dried extract. The chloroform extract evaluated for phenolic contents showed the values of 5.01 ± 0.90 mg GAE/g of dried extract. The hexane extract of *G.trichophylla* showed the lowest values of phenolic contents with 3.214 ± 0.35 mg GAE/g of dried extract. The flavonoids contents of *G.trichophylla* extracts were expressed as quercetin equivalents in mg QE/g of dry weight. The flavonoids contents for *G.trichophylla* extracts of methanol, chloroform and hexane were found to be 41.345 ± 0.19, 9.828 ± 0.78, and 26.793 ± 1.45 mg QE/g, respectively Table 1.

**Table 1:** Comparison of Phenolic, flavonoids contents, DPPH, FRAP antioxidant assays, and Lipoxygenase inhibition of *Gaultheria trichophylla* extracts

Samples	Phenolic contents mg GAE/g of extract	Flavonoids contents mg QE/g of extract	DPPH % inhibition 0.5 mg/ml	IC_50_ μg/ml	FRAP % inhibition 0.5 mg/ml	IC_50_ μg/ml	5- LOX Inhibition	
							% Inhibition at 0.5 mg/ml	IC_50_ μg/ml
G. MeOH	17.5 ± 2.5	41.3 ± 0.1	90.5 ± 0.8	81.2 ± 0.2	98.2 ± 1.7	11.2 ± 0.1	90.5 ± 0.7	277.3 ± 2.7
G. Chlor	5.0 ± 0.9	9.8 ± 0.7	66.8 ± 0.6	119.2 ± 0.7	77.0 ± 2.7	27.7 ± 0.6	66.9 ± 0.1	379.3 ± 1.4
G. Hex	3.2 ± 0.3	26.7 ± 1.4	58.9 ± 0.2	99.0 ± 0.5	74.2 ± 1.4	13.5 ± 0.7	57.0 ± 0.3	448 ± 0.2
Quercetin	-	-	92.1 ± 0.04	16.9 ± 0.1	95.2 ± 0.1	6.04 ± .003	-	-
Vitamin C	-	-	93.1 ± 0.06	13.5 ± 0.1	94.3 ± 0.09	5.82 ± 0.02	-	-
Baicalein	-	-	-	-	-	-	95.7 ± 1.2	22.4 ± 1.3

Values are given as standard error mean (SEM±) of three determination

**Fig. 1:**
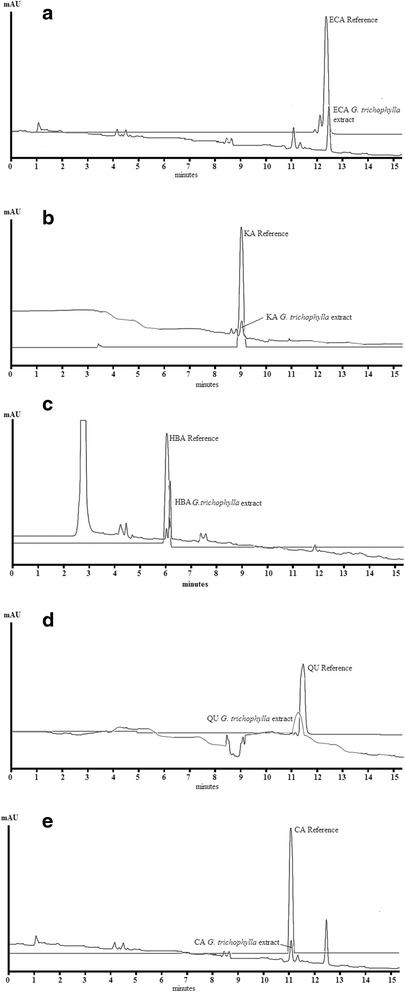
**a-e** Qualitative HPLC analysis of extract of *G. trichophylla* with standard phenolics, **a** = epicatechin (ECA), **b** = kaemferol (KA), **c** = hydroxyl benzoic acid (HBA), **d** = quercetin (QU) and catechin (CA)

The qualitative HPLC analysis of crude methanol extract of *G. trichophylla* revealed the presence of phenolic compounds including hydroxyl benzoic acid (HBA) with retention time (RT) of 6.2 min when the compound was scanned at 250 nm wavelength (λ). The other compounds detected were, kaemferol (KA); RT = 9.1, λ = 310, catechin (CA); RT = 11.1, λ = 280, epicatechin (ECA); RT = 12.5, λ = 280, and quercetin (QU); RT = 11.5, λ = 360. Fig. 1a-e.

**Fig. 2:**
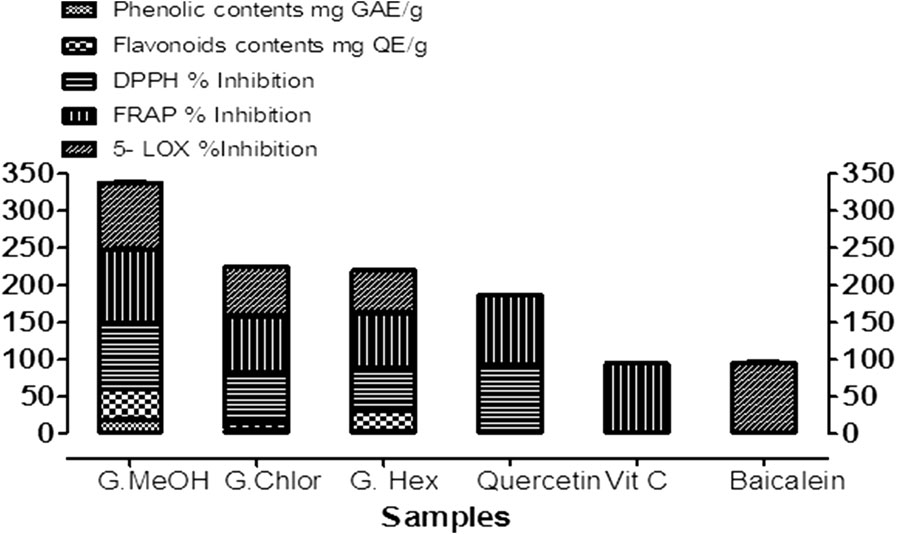
Comparison of phenolic, flavonoids contents, DPPH, FRAP assays, and Lipoxygenase inhibition of *Gaultheria trichophylla* extracts

### Determination of antioxidant activities

DPPH assay was carried out to investigate the antioxidant potential of *G. trichophylla*. The methanol extract of *G.trichophylla* showed the maximum inhibition of 90.55 ± 0.89% with IC_50_ = 81.2 ± 0.26 μg/ml. The chloroform extract showed the inhibition of 66.83 ± 0.64% with IC_50_ = 119.2 ± 0.7 μg/ml. The hexane extract showed the inhibition of 58.97 ± 0.23% with IC_50_ = 99.03 ± 0.5 μg/ml. All the results were compared with Quercetin and Vitamin C as standard antioxidants. The other antioxidant assay conducted was FRAP assay to investigate the antioxidant activity of *G. trichophylla*. The methanol extract of *G.trichophylla* showed the maximum inhibition of 98.28 ± 1.71% with lowest IC_50_ = 11.2 ± 0.16 μg/ml. The chloroform extract showed the inhibition of 77.09 ± 2.7% with IC_50_ = 27.7 ± 0.6 μg/ml. The hexane extract showed the inhibition of 74.28 ± 1.4% with IC_50_ = 13.5 ± 0.7 μg/ml Table 1.

### Anti-lipoxygenase activity

The *G. trichophylla* methanol extract showed the maximum lipoxygenase inhibition with 90.50 ± 0.7% followed by chloroform extract 66.98 ± 0.14% and hexane extract 57.05 ± 0.33%.

All the results are tabulated in Table 1 and are expressed as comparison in Fig. 2.

### Minerals and heavy metal contents

Determinations of mineral contents of *Gaultheria trichophylla* are presented in Table 2. The most abundant minerals found were magnesium (4.115 mg/100 g) and potassium (1.935 mg/100 g) and least abundant was copper (0.00097 mg/100 g). The concentration of Ni and Co in *G. trichophylla* sample were 0.162 and 0.043 ppm respectively, which are much less in concentration. The concentration of mercury (0.76 ppm) in sample was also very low as compared to allowed limits (0.8 ppm) Table 2.

**Table 2:** The concentrations of minerals and heavy metal contents in *G. trichophylla*

No	Minerals	Contents (mg/100 g)	Metals	Concentration (ppm)
1	Na	0.3186 ± 0.02	Ni	0.162 ± 0.017
2	Ca	0.2191 ± 0.011	Pb	0.649 ± 0.02
3	Mg	4.115 ± 0.012	Hg	0.76 ± 0.24
4	Fe	0.089 ± 0.001	Si	1.574 ± 0.04
5	K	1.935 ± 0.011	Co	0.043 ± 0.012
6	Zn	0.0128 ± 0.002	Cr	1.277 ± 0.02
7	Mn	0.0118 ± 0.01	As	00.01 ± 0.19
8	Cu	0.00097 ± 0.0001	Cd	0.177 ± 0.015

Values are given as standard error mean (SEM±) of three determination

## Discussions

Plants are used traditionally for various conditions in many system of medicine. The claim of their use for different ailments needs to be justified. The phenolic and flavonoids contents of plants are very important medicinally. They are mostly considered as compounds responsible for antioxidant potential and therefore are evaluated mostly for the said purpose. It is now well known that the antioxidant activities of phenolic contents are mainly due to their ability to scavenge radical, donate hydrogen and quench the singlet oxygen [34]. There are many reports that the antioxidant activities of plants are due to presence of phenolic compounds [35].

These phenolic compounds can be used as possible quality control indicators in herbal products. Our previous HPLC analysis for phenolic compounds also showed the presence of phenolic compounds in extracts of *G.trichophylla* [17].

The extracts of *G.trichophylla* showed the presence of phenolic and flavonoids and most of these are concentrated in the methanol extract. This may have contributed towards its greatest radical scavenging activity in both DPPH and FRAP assays. The principle of this method is based on the reduction of purple colored DPPH solution in the presence of hydrogen donating antioxidants by the formation of yellow colored diphenyl-picryl hydrazine. DPPH free radical scavenging assay is more indirect assay [36]. The DPPH activity was significant for methanol extract of *G.trichophylla*. FRAP assay proved to be much sensitive comparatively and showed more inhibition of extracts tested.

The extraction of compounds from plants with antioxidant potential is often carried out using the organic solvents like methanol, chloroform and hexane etc. There is always variation in antioxidant activities of different plant samples extracted with different organic solvents which is attributed to the presence of variety of secondary metabolites [37]. And this can be observed in case of *G. trichophylla*, where extracts with different polarities of solvents showed different antioxidant activities. The methanol extract showed the maximum inhibition in DPPH and FRAP assay. This showed that most of the phenolic compounds are concentrated in the most polar solvent used for extraction as evident in phenolic content and flavonoids assay.

The analysis of correlation between the total phenolic/flavonoids contents and antioxidant and anti-inflammatory (LOX inhibition) activities showed significant dependence as revealed in the Fig. 2. The methanol extract of *G.trichophylla* inhibited the enzyme lipoxygenase significantly and showed very good potential of being lipid lowering and possibly anti-inflammatory agent. Chloroform and hexane extracts also showed good to moderate activity against the enzyme.

Every mineral analyzed have its own importance in human body. Magnesium have a very important role in structure and function of the human body [38]. *Gaultheria trichophylla* presented high magnesium contents. It is reported that high potassium contents in the body enhance the utilization of iron and in patient suffering from extra excretion of potassium from the body fluid and are using diuretics to control hypertension [39]. To maintain the electrolyte balance in the body sodium has a very important part. Calcium is an important mineral and has beneficial effect in development of teeth and bones in children, pregnant and lactating women. It is well known that Iron is required in the body for the formation hemoglobin and a person can get anemic in case of deficiency. Manganese (Mn) is known as an essential trace element which acts as cofactor for many enzymes. Zinc (Zn) is an essential component of thousands of proteins in plants, although it is toxic in excess quantities [32]. Similarly other minerals also play their role in functioning of human body. It is clear from the analysis that *G. trichophylla* beside its medicinal values is also important in nutritional point of view.

Besides the wholesome minerals, the contents of heavy metals (As, Cd, Ni, Cr, Hg and Pb) are also the important standard to identify the quality of the plant. The medicinal plants may face serious consequences if heavy metal accumulation beyond permissible limits is detected. Therefore, it is necessary to check the levels of pollutants in extracts of medicinal plants before use. This practice of standardization will help to select the proper site of collection of medicinal plants and will exclude the environmentally polluted sites. The contents of heavy metals are defined by certain limits and these limits are different in different areas of the world e.g. the minimum permissible limits for arsenic, lead, cadmium, mercury and chromium are 5.0, 10.0, 0.3, 0.2 and 2.0 ppm respectively in Canada respectively [40]. The results showed that the concentrations of heavy metals in *G. trichophylla* were either low or are within defined limits [40]. Chromium (Cr), is considered one of the most toxic pollutant element. The permissible limit for Cr in raw herbal materials is 2.0 ppm and that for finished products is 0.02 mg/day [40]. Another element, Lead (Pb) is highly toxic for plants, animals and microorganisms which escalate in pollution due to increased fertilizer consumption, fuel combustion and sewage sludge. The sample of *G.trichophylla* showed to contain low concentration of Pb (0.649 ppm) as compared to the permissible limit of 10 ppm defined by [40]. Cadmium (Cd) is which occurs widely in medicinal plants is a hazardous heavy metal. The major sources leading to accumulation of cadmium in soil and plants are phosphate fertilizers. The sample of *G. trichophylla* analyzed in this study had Cd concentration (0.177 ppm) within the acceptable range of 0.3 ppm recommended by [40]. Ni is considered as allergen and has direct interaction with proteins and Co is mostly used as component of cyanocobalamin vitamin [41]. However no limit has been set for Nickel food stuffs [42]. The concentration of Co, another major cause of contact dermatitis next only to nickel and chromium, was also found to be within permissible limit [43].

## Conclusion

In our experiments we have evaluated the *G.trichophylla* extracts for antioxidant and lipoxygenase activities. The results revealed that methanol extract of *G.trichophylla* is more concentrated with the phenolic and flavonoids contents and, therefore, have more DPPH and FRAP reducing activities. The results clearly indicate that *G.trichophylla* has strong antioxidant and anti-inflammatory potential and can be a source of important alternative therapeutic agent. Moreover, investigation also revealed that *Gaultheria trichophylla* is fortified with important minerals and it is a safe as a food and drug. As far environmental contaminants are concern it grows in hilly areas with very less population effect.

## Highlights

- *Gaultheria trichophylla* is an important medicinal plant.
- Phenolic compounds are related to antioxidant activities.
- Inhibition of 5-LOX showed its anti-inflammatory potential.
- Investigation showed its nutritional importance.
- In crude form it is a safe drug to use.

## Abbreviations

DPPH: 2,2-diphenyl-1-picrylhydrazyl
FRAP: Ferrous reducing antioxidant power
G. trichophylla: Gaultheria trichophylla
GAE: Gallic acid equivalent
HPLC: High performance liquid chromatography
LOX: Lipoxygenase
TPC: Total phenolic contents
